# Factors Associated with Severe Presentation and Mortality in ICU-Acquired Candidaemia: A 20-Year Single-Centre Retrospective Cohort

**DOI:** 10.3390/jcm15218597

**Published:** 2026-11-04

**Authors:** Ayse Sena Sisman, Ugur Onal, Busra Calisir, Emel Yilmaz, Esra Kazak, Yasemin Heper, Guven Ozkaya, Remzi Iscimen, Cuneyt Ozakin, Beyza Ener, Halis Akalin

**Affiliations:** 1 Department of Infectious Diseases and Clinical Microbiology, Faculty of Medicine, Bursa Uludag University, Bursa 16059, Türkiye; aysesena1629@gmail.com (A.S.S.); uguronal@uludag.edu.tr (U.O.); emelyilmaz@uludag.edu.tr (E.Y.); eskazak@uludag.edu.tr (E.K.); yheper@uludag.edu.tr (Y.H.); 2 Department of Medical Microbiology, Faculty of Medicine, Bursa Uludag University, Bursa 16059, Türkiye; busracalisir@uludag.edu.tr (B.C.); ozakin@uludag.edu.tr (C.O.); bener@uludag.edu.tr (B.E.); 3 Department of Biostatistics, Faculty of Medicine, Bursa Uludag University, Bursa 16059, Türkiye; ozkaya@uludag.edu.tr; 4 Department of Anesthesiology and Reanimation, Faculty of Medicine, Bursa Uludag University, Bursa 16059, Türkiye; remdefne@uludag.edu.tr

**Keywords:** candidaemia, intensive care unit, mortality, antifungal treatment, fluconazole, echinocandins

## Abstract

**Background/Objectives:** The risk factors for the severe presentation (severe sepsis or septic shock) are not fully understood in ICU-acquired candidaemia. The primary objective of this study was to evaluate risk factors for the severe presentation (severe sepsis or septic shock) and mortality in patients with candidaemia in ICUs. Secondarily, we compared 28-day mortality between patients treated with fluconazole and those treated with an echinocandin. **Methods:** A retrospective cohort design was employed in a single-center ICU setting, encompassing a study period from 2004 to 2023. Adult patients who had *Candida* growth in blood cultures taken 48 h after admission to the ICU were included in the study. Risk factors for the severe presentation (severe sepsis and septic shock) and mortality were evaluated in the 141 patients included in the study. The treatment comparison between fluconazole and echinocandins was performed in 113 patients (with 48-h rules) and 125 patients (without 48-h rules). **Results:** Among 141 patients, 77 (54.6%) died within 28 days. We found that in the multivariable model, APACHE II score, TPN use, steroid use, and concomitant bacteremia were associated with severe presentation. Each one-point increase in APACHE II score was associated with higher risk of severe presentation (adjusted OR 1.146, 95% CI 1.056–1.242; *p* = 0.001). TPN use and steroid use were also associated with higher risk (adjusted OR 3.297, 95% CI 1.265–8.590; *p* = 0.015; and adjusted OR 6.067, 95% CI 1.486–24.768; *p* = 0.012, respectively). Concomitant bacteraemia was associated with lower odds of severe presentation. Patients with severe presentation (severe sepsis or septic shock) had a higher risk of 28-day mortality than patients with sepsis (adjusted OR 8.06, 95% CI 2.60–25.00; *p* < 0.001). In the primary cohort (n = 113; overlap weights), 28-day mortality was 49.2% with fluconazole and 52.1% with echinocandin (OR 1.12, 95% CI 0.37–3.45; *p* = 0.838). Results were consistent in the 125-patient cohort without the 48-h rule (OR 1.40, 95% CI 0.51–3.81; *p* = 0.508). **Conclusions:** Each one-point increase in APACHE II score on admission, TPN use, and steroid use was associated with severe presentation in ICU-acquired candidaemia. The severity of the clinical presentation at the onset of candidaemia (presence of severe sepsis or septic shock) was strongly associated with 28-day mortality. No difference in 28-day mortality was detected between fluconazole and echinocandin treatment, but this comparison was imprecise and cannot inform antifungal choice.

## 1. Introduction

In recent years, invasive fungal infections have been on the rise due to increasing comorbidities and immunosuppressive treatments. It has been reported that approximately 70% of all invasive fungal infections are caused by *Candida* [1,2,3]. Mortality rates in invasive candidiasis range from 5% to 71%, with crude mortality estimated at approximately 40% [4,5]. This rate, which reaches 60% in intensive care units (ICUs), has been reported to exceed 80% in patients with candidaemia and septic shock [6]. The presence of septic shock at the onset of candidaemia, along with high APACHE-II and SOFA scores, has been shown to increase mortality [5,7]. Total parenteral nutrition (TPN), liver failure, renal failure and advanced age have been identified as risk factors in various studies [8,9]. In the European Confederation of Medical Mycology (ECMM) Candida III European Observational Cohort Study, attributable mortality was found to be 18.2%. The same study emphasised that the most important risk factor for mortality was admission to the ICU [5]. Mortality rates vary depending on the severity of clinical condition at the onset of candidaemia, early appropriate antifungal treatment, source control, and the type of *Candida*. Delays in antifungal treatment and failure to control the source early on have been reported as other important factors affecting mortality [10,11,12]. The primary objective of this study was to evaluate risk factors for the severe presentation (severe sepsis or septic shock) and mortality in patients with candidaemia in our ICUs. Secondarily, we compared 28-day mortality between patients treated with fluconazole and those treated with an echinocandin.

## 2. Materials and Methods

This study included adult (≥18 years) patients who developed ICU-acquired candidaemia in our hospital between 2004 and 2023. Permission for the study was obtained from the Clinical Research Ethics Committee of Bursa Uludağ University Faculty of Medicine on 3 May 2023, with decision number 2023–10/4. Since the study data were completely anonymous and the study was retrospective, the requirement for patient informed consent was waived by the Institutional Review Board.

Bursa Uludağ University Faculty of Medicine Hospital is a 900-bed tertiary university hospital accredited by the Joint Commission International in 2007 and 2012.

Patients with *Candida* spp. growths in blood cultures who were followed up in adult ICUs (Anaesthesiology-Reanimation ICU, General Surgery, Neurosurgery, Thoracic and Cardiovascular Surgery, and Cardiology) were retrospectively screened from our hospital‘s mycology laboratory archive. Demographic and clinical data of the patients were obtained from medical records in the hospital electronic file system.

Adult patients with *Candida* spp. growth in blood cultures taken 48 h after admission to the ICU (ICU-acquired) were included in the study; the exclusion criteria were mixed growth (growth of different *Candida* species in consecutive blood cultures obtained within 7 days), a diagnosis of COVID-19, inaccessible electronic data, and those under the age of 18 (Figure 1). In patients with multiple candidaemia episodes, the first episode was included in the study.

The evaluation was performed on 141 patients, while the following rules were taken into account in the treatment evaluation. Patients who survived for at least 48 h after blood culture was taken and received antifungal treatment for at least 48 h were included in the analysis. According to these rules, the treatment comparison between fluconazole and echinocandins was performed in 113 patients (Figure 1). Treatment comparison between fluconazole and echinocandins was also performed in 125 patients (patients received fluconazole or echinocandins) without the 48 h rule.

Early treatment was defined as antifungal therapy administered within the first 48 h following blood culture collection. Treatment evaluation was performed on the fluconazole and echinocandin groups (other antifungal treatments were not included in the statistical calculations). It was observed that 10 patients underwent antifungal change after receiving empirical antifungal treatment for at least three days. Therefore, these patients were included in the antifungal treatment group they initially received.

## 3. Definitions

Candidaemia: Defined as *Candida* spp. growth in at least one blood culture. The day the blood culture was taken was considered the first day of candidaemia.

Candidaemia developing in the ICU (ICU-acquired candidemia): Patients who had *Candida* spp. growth in blood cultures taken 48 h after admission to the ICU (Candidaemia developing 48 h after the patient’s admission to the ICU) [13].

Adult patient: Patients aged ≥18 years.

Neutropenia: Defined as an absolute neutrophil count of <500/mm^3^ in a complete blood count [14].

Prolonged steroid use: Defined as the use of prednisolone or equivalent steroids at a dose of >20 mg/day for more than three weeks [15].

Pulse steroid use: Defined as the intravenous (IV) administration of 250 mg of prednisolone or equivalent steroids for one or more days [16].

Immunomodulatory therapy: Defined as molecules that affect the immune system by exerting stimulatory, suppressive, or regulatory effects on the immune system [17].

ECMM quality of clinical candidaemia management (known as EQUAL) *Candida* score: A scoring system developed by the ECMM to measure compliance with guidelines in the management of candidaemia [18,19]. It consists of a total of 10 criteria under the steps of diagnosis, treatment, and follow-up (Table 1).

Candidaemia developing under antifungal treatment (breakthrough candidaemia): This is defined as *Candida* spp. growth in patients who have been receiving antifungal treatment for at least 72 h at the onset of candidaemia [20].

*Candida* spp. colonization was assessed via clinical cultures rather than routine surveillance.

Concomitant bacteraemia: This was assessed by screening blood cultures taken within 48 h prior to the onset of candidaemia.

Empirical treatment: This is defined as treatment initiated upon suspicion of candidaemia, while targeted treatment is defined as treatment initiated following *Candida* spp. growth in blood cultures. Patients with breakthrough candidaemia were excluded from the empirical or targeted treatment evaluation.

Central venous catheter-associated candidaemia: Defined as the growth of the same pathogen in blood cultures obtained from both the peripheral venous route and the central venous catheter [20].

Intra-abdominal candidaemia: Defined as the presence of any of the following conditions [21,22]:

- presence of primary/secondary/tertiary peritonitis,
- growth of *Candida* spp. in cultures taken from the abdomen during abdominal surgery,
- presence of perforation/leakage in the bile ducts or gastrointestinal tract, or
- presence of the same pathogen in cultures taken from drains placed within the first 24 h after surgery.

Urinary-related candidaemia: Defined as the growth of the pathogen in blood cultures after the procedure, which was present in urine cultures prior to urological instrumentation [23].

Primary candidaemia or potential primary sources of infection: Patients who did not fit into any of the three groups or whose source of candidaemia could not be clearly identified (presence of *tracheoesophageal fistula*; skin-soft tissue, deep tracheal aspirate, urinary colonisation, etc.).

To better assess the clinical status of patients and mortality associated with candidaemia, APACHE-II scores were calculated on the first day of intensive care admission. The clinical picture on the day of candidaemia was evaluated using Sepsis-1 criteria. According to these criteria, while sepsis represents a hemodynamically stable condition (absence of hypotension, non-severe presentation), severe sepsis and septic shock are characterized by the presence of hypotension and hemodynamic instability (severe presentation) [24]. Survival data after hospital discharge (at 3 and 6 months) were ascertained by cross-referencing patient identity numbers with the National Death Registry database.

## 4. Microbiological Methods

Blood cultures obtained from patients were incubated for five days in the BD BACTEC FX top (Becton-Dickinson and Company™, Franklin Lakes, NJ, USA) automated blood culture system. Gram staining was performed when a growth signal was detected in the blood culture during the incubation period. Concurrently, passages were made to 5% sheep blood agar medium and eosin methylene blue (EMB; BD) medium and incubated at 37 °C for 24–48 h. Additionally, cultures with Gram staining morphology resembling yeast were also inoculated onto Sabouraud dextrose agar (BD) containing gentamicin and chloramphenicol.

Yeast colonies growing on the inoculated media were first identified using conventional methods (germ tube test, cornmeal tween 80, etc.). In addition to these methods, isolates were also identified using API ID 32 C (BioMérieux, Marcy l’Etoile, France) until 2020 and matrix-assisted laser desorption ionisation time-of-flight mass spectrometry (MALDI TOF MS; Bruker Daltonics, Bremen, Germany) from 2020 to the present.

The in vitro susceptibilities of the isolates to fluconazole, voriconazole, itraconazole, posaconazole, amphotericin B, micafungin and anidulafungin were determined using the microdilution method recommended in the CLSI M27 guideline. The minimum inhibitory concentration data obtained were interpreted using species-specific epidemiological breakpoint values or clinical cutoff values according to the CLSI supplement M27M44S document [25,26].

## 5. Statistics

The normality of the data distribution was examined using the Shapiro–Wilk test. Descriptive statistics were presented as mean and standard deviation (minimum-maximum) values for quantitative data, and as frequency and percentage for qualitative data. For independent two-group comparisons, the *t*-test was used for data showing a normal distribution, and the Mann–Whitney U test was used for variables not showing a normal distribution. Pearson’s chi-square, Fisher–Freeman–Halton, and Fisher’s exact chi-square tests were used for the analysis of categorical data. Variables associated with severe sepsis or septic shock in patients with candidaemia were evaluated using binary logistic regression analysis. The significance level was set at α = 0.05. Statistical analysis of the data was performed using IBM SPSS 29.0.2.0 (IBM Corp. Released 2023. IBM SPSS Statistics for Windows, Version 29.0.2.0, Armonk, NY, USA).

To reduce confounding by indication in the comparison of fluconazole and echinocandin, we used propensity score weighting. The propensity score for receiving an echinocandin as first-line therapy was estimated by logistic regression including age, sex, APACHE-II score at ICU admission, severe presentation (severe sepsis or septic shock) on the day of candidaemia, continuous renal replacement therapy, chronic renal failure, diabetes mellitus, at least one immunosuppressive condition, Candida albicans versus non-albicans species, and source of candidaemia (abdominal, central venous catheter, or primary/other). These covariates were selected a priori as factors that may influence both the choice of antifungal agent and mortality. Stabilized inverse probability of treatment weights (IPTW) were calculated first. Because echinocandins were given almost exclusively to patients with non-albicans species, conventional IPTW produced extreme weights and residual imbalance; therefore, overlap weights were used as the primary weighting method on the basis of these balance diagnostics. Overlap weights assign each patient a weight proportional to the probability of receiving the alternative treatment, which emphasizes patients for whom either agent was a plausible choice and yields exact mean balance on the covariates included in the logistic propensity model. Covariate balance was assessed using standardized mean differences (SMD), with an absolute SMD <0.10 considered adequate, and the effective sample size of each group was reported. The association between treatment and 28-day mortality was estimated by weighted logistic regression and expressed as an odds ratio (OR), and the absolute risk difference was estimated by a weighted linear probability model; 95% confidence intervals (CIs) were based on robust (sandwich) standard errors. The primary analysis included the 113 patients who survived and received antifungal therapy for at least 48 h. Sensitivity analyses included conventional stabilized IPTW, IPTW truncated at the 1st and 99th percentiles, and all three weighting methods repeated in the 125 patients who received fluconazole or an echinocandin as first-line therapy, regardless of the 48 h rule. The robustness of the findings to unmeasured confounding was assessed by E-values. Propensity score analyses were performed in R version 4.3.3 (R Foundation for Statistical Computing, Vienna, Austria) using the survey, sandwich, and lmtest packages.

## 6. Results

A total of 141 patients were included in the study. Of these, 71 (50.3%) were male, and 70 (49.7%) were female. The mean age was 56.2 ± 18.4 (range 19–95). The mean total hospital stay and mean ICU stay were 30.9 ± 37.4 (2–321) days and 20.6 ± 34.4 (2–321) days, respectively. The average APACHE-II score at admission to the ICU was 23.67 ± 7.92 (4–46). A total of 63,944 patient admissions to ICUs were observed during the study period. The incidence of eligible analyzed candidaemia per 1000 patient admissions was 2.2, and the incidence of eligible analysed candidaemia per 10,000 patient days was 4.1.

Demographic, epidemiological and clinical characteristics of patients are shown in Table 2. No patients were diagnosed with endocarditis in the examinations performed.

Risk factor assessment for the severe presentation (severe sepsis or septic shock) in candidaemia cases was performed in the 141 patients (Table 3). When patients who had sepsis were compared with those who had severe sepsis or septic shock, the presence of multiple trauma was significantly lower in the severe sepsis or septic shock group (*p* = 0.047); age, APACHE-II score at ICU admission, TPN, steroid use, presence of coronary artery disease and presence of at least one condition causing immunosuppression were significantly higher in patients who had severe sepsis or septic shock (*p* = 0.011, *p* < 0.001, *p* = 0.004, *p* = 0.042, *p* = 0.002 and *p* = 0.048, respectively) (Table 3).

In the multivariable model, APACHE II score, TPN use, steroid use, and concomitant bacteremia were associated with severe presentation. Each one-point increase in APACHE II score was associated with higher risk of severe presentation (adjusted OR 1.146, 95% CI 1.056–1.242; *p* = 0.001). TPN use and steroid use were also associated with higher risk (adjusted OR 3.297, 95% CI 1.265–8.590; *p* = 0.015; and adjusted OR 6.067, 95% CI 1.486–24.768; *p* = 0.012, respectively). Concomitant bacteraemia was associated with lower odds of severe presentation (adjusted OR, 0.205; 95% CI, 0.067–0.624; *p* = 0.005). Age, multitrauma, and coronary artery disease were not statistically significantly associated with severe presentation.

Among 141 patients, 77 (54.6%) died within 28 days. In the univariable analyses, APACHE II score, Equal Candida score, severe sepsis or septic shock, total parenteral nutrition, chemotherapy/radiotherapy, multitrauma and early antifungal treatment were associated with 28-day mortality (Table 4). In the multivariable model, patients with the severe sepsis or septic shock had a higher risk of 28-day mortality than patients with sepsis (adjusted OR 8.06, 95% CI 2.60–25.00; *p* < 0.001).

Among the 131 patients who received antifungal treatment, 28-day mortality was 62.8% (49/78) in the early-treatment group and 34.0% (18/53) in the delayed-treatment group. The crude association between early treatment and mortality was statistically significant (OR 3.285, 95% CI 1.582–6.823; *p* = 0.001). However, this association was attenuated after adjustment for APACHE II score and sepsis severity (adjusted OR 2.183, 95% CI 0.951–5.012; *p* = 0.066) and was not statistically significant in the fully adjusted model (adjusted OR 2.030, 95% CI 0.835–4.934; *p* = 0.118). In analyses stratified by sepsis severity, mortality was higher in the early-treatment group both among patients with sepsis without severe sepsis or septic shock (23.1% vs. 8.7%; OR 3.15, 95% CI 0.45–21.95; *p* = 0.328) and among patients with severe sepsis or septic shock (70.8% vs. 53.3%; OR 2.12, 95% CI 0.87–5.18; *p* = 0.110). Neither stratified association was statistically significant.

The comparison of 113 patients treated with fluconazole or echinocandin is shown in Table 5. The rates of at least one immunosuppressive condition, chronic renal failure, diabetes mellitus, continuous renal replacement therapy, and the proportion of non-*C. albicans* species were significantly higher in the echinocandin group (respectively; *p* = 0.024, *p* = 0.014, *p* = 0.028, *p* = 0.001 and *p* = 0.001); however, no significant difference was found between the two groups in terms of severe presentation (presence of severe sepsis or septic shock at the onset of candidaemia, APACHE-II score at ICU admission) and mortality.

Before weighting, patients treated with an echinocandin more often had continuous renal replacement therapy (40.0% vs. 5.7%), chronic renal failure (24.0% vs. 5.7%), diabetes mellitus (36.0% vs. 15.9%), and an immunosuppressive condition (60.0% vs. 38.6%), and less often had *C. albicans* candidaemia (4.0% vs. 46.6%) (Supplementary Table S1). Conventional stabilized IPTW did not achieve adequate balance: the effective sample size of the echinocandin group fell from 25 to 7.1, and the largest absolute SMD remained 0.89 (Supplementary Table S2). Overlap weighting achieved an exact balance across all covariates (all SMDs 0.00), with an effective sample size of 18.8 in the echinocandin group. In the overlap-weighted analysis, 28-day mortality was 49.2% in the fluconazole group and 52.1% in the echinocandin group (OR 1.12, 95% CI 0.37–3.45; *p* = 0.838; risk difference 2.9 percentage points, 95% CI −25.1 to 30.9) (Table 6). Conventional and truncated IPTW yielded larger point estimates with wider confidence intervals (OR 2.18, 95% CI 0.53–9.03; OR 1.84, 95% CI 0.49–6.92, respectively). When the analysis was repeated in the 125 patients who received either agent regardless of the 48-h rule, results were consistent (overlap-weighted OR 1.40, 95% CI 0.51–3.81; *p* = 0.508). All 12 patients excluded by the 48-h rule (seven treated with an echinocandin and five with fluconazole) died within 28 days. E-values for the point estimates ranged from 1.31 to 2.31; because all confidence intervals included 1, the E-value for the confidence limit was 1.00 across all analyses.

## 7. Discussion

According to our findings, the 28-day mortality rate was determined to be 54.6% in an evaluation of 141 patients. In ICU studies conducted in our country, the 30-day mortality rate has been reported to be between 36.7% and 83% [27,28,29,30,31]. In international and national multicentre studies, 30-day mortality rates have been reported to range between 40.6% and 52.4% [8,32,33,34,35,36]. Mortality rates vary from centre to centre depending on the patient population receiving healthcare services, as well as the predominant *Candida* spp. and resistance rates, and they appear across a wide spectrum [37].

It is known that mortality rates are higher in patients with invasive candidiasis in septic shock, with rates ranging from 54% to 87% [8,38,39]. While many studies have shown that clinical severity (presence of severe sepsis or septic shock, high sequential organ failure assessment [SOFA]/[acute physiology and chronic health evaluation II] APACHE-II score) affects mortality [40,41,42,43], the risk factors for the severe presentation (severe sepsis or septic shock) in patients with candidaemia are not fully understood [39,43]. In our study, TPN use, higher APACHE-II score at ICU admission, and steroid use were independently associated with severe presentation (severe sepsis or septic shock).

It is known that TPN use creates immunosuppression by triggering hyperglycaemia, hyperinsulinemia, metabolic stress, and inflammation, which are risk factors for severe sepsis. In addition, the heightened potential for translocation occurring as a result of increased *Candida* colonization is another factor that affects clinical severity [44,45,46]. Alternatively, these patients might have received TPN secondary to major abdominal surgery, potentially leading to catheter-associated candidaemia via the central venous line utilized for TPN administration. Consequently, these clinical variables may have functioned as residual confounders in the present analysis.

The fact that a high APACHE-II score, one of the prognostic scoring systems used for patients monitored in an ICU, is an independent risk factor for severe sepsis or septic shock can be explained by the relationship between high proinflammatory cytokine levels and a high APACHE-II score and, consequently, a severe clinical picture. In particular, it has been shown that elevated IL-6 levels correlate with high APACHE-II scores and increase the risk of organ failure and, consequently, severe sepsis [47,48].

Corticosteroid use was associated with severe presentation. A biologically plausible explanation is the effect of glucocorticoids on neutrophil function and cytokine responses, although our data cannot establish this mechanism. Primarily, glucocorticoids impair neutrophil chemotaxis, phagocytosis, and intracellular killing mechanisms, which constitute the frontline defense against *Candida* species. Furthermore, steroids downregulate the transcription of critical pro-inflammatory cytokines (such as TNF-α, IL-1, and IL-6) and disrupt the Th1/Th17 immune response axis, leading to uncontrolled fungal dissemination [49].

Concomitant bacteraemia was associated with lower odds of severe presentation. This finding should not be interpreted as a protective effect; it may reflect earlier recognition and more intensive management of patients with documented bacteraemia, or residual confounding. While this finding appears counterintuitive, it most likely reflects clinical management dynamics rather than a biological advantage. Patients with documented or suspected bacterial bloodstream infections are routinely managed with aggressive broad-spectrum empirical antimicrobial therapies and undergo prompt source control, such as the rapid removal of central venous catheters. This heightened clinical suspicion and timely therapeutic intervention may inadvertently halt the progression of candidaemia toward septic shock.

In our study, the benefit of early antifungal treatment on survival could not be demonstrated. In univariate analysis, early antifungal treatment was identified as a factor increasing the risk of death among the factors affecting survival. The APACHE-II score on the day of ICU admission was 24.1 ± 7.3 (5–42) in the group that started early antifungal treatment and 20.4 ± 6.9 (4–37) in the group that started late treatment (*p* = 0.006). Additionally, the number of patients with severe sepsis or septic shock was 53 (80%) in the early treatment group, while it was 27 (54%) in the late treatment group (*p* = 0.002). These results can be explained by the initiation of early antifungal treatment in patients due to clinical severity and the high mortality risk associated with current clinical severity, when considering previous studies. This situation is similar to studies showing that clinical severity (presence of septic shock or severe sepsis at the onset of candidaemia) is the main factor determining mortality. Although it is known that early antifungal treatment reduces mortality in candidaemias [11,50], there are also studies that fail to demonstrate this relationship [12,51,52]. Additionally, the proportion of ICU-acquired candidaemia studies evaluating early antifungal treatment is relatively low, and studies evaluating the effect of early antifungal treatment in candidaemia generally have heterogeneous populations [12,53,54]. In a prospective study conducted in Australia by Marriot et al. [51], it was reported that mortality in candidaemia cases in non-neutropenic patients was associated with the patient‘s clinical severity rather than the type of antifungal agent or timing of treatment. Similarly, in a three-year retrospective intensive care study by Bienvenu et al. [55], the time to initiation of antifungal treatment was significantly shorter in deceased patients, and these patients had higher SOFA and simplified acute physiology score II scores compared to surviving patients. In an ICU study by Ohki et al. [20], the effect of early removal of the central venous catheter or early antifungal treatment on mortality could not be demonstrated. Fernandez et al. [52] reported no significant difference in the time to initiation of antifungal treatment between survivors (61 h) and non-survivors (59 h) in candidaemia, Morrell et al. [11] found increased mortality rates in patients treated after the 12th hour of candidaemia; however, this study also emphasised that only nine patients received antifungal treatment within 12 h, and there was no progressive increase in mortality in patients treated after 12 h.

In our study, the presence of severe presentation (severe sepsis or septic shock) was identified as an independent risk factor increasing the risk of death. In three different studies conducted by Bassetti et al., one of which was prospective [43], it was shown that the presence of septic shock in patients with candidaemia increases mortality [8,38]. In addition to the presence of septic shock, the presence of severe sepsis has also been shown to increase mortality, and in candidaemia cases developing in the ICU, the severity of the disease or the presence of organ failure is known to be more important than the type of Candida and its susceptibility [12,35].

In our study, we compared 28-day mortality between fluconazole and echinocandin treatment. The wide confidence intervals are compatible with clinically important benefit or harm of either agent; therefore, the absence of a statistically significant difference should not be interpreted as evidence of equivalence.

A multicentre retrospective study from Spain [56] showed that echinocandins were a protective factor in terms of 30-day mortality, and a prospective study of 51 patients from France also showed that echinocandins were associated with survival [57]. However, in another study from France, no statistically significant difference was found between fluconazole and echinocandin use in terms of mycological success at 30 days and mortality at 30 and 90 days. The same study showed that a high SOFA score on the day of candidaemia and inadequate source control were independent risk factors for mortality [55]. Another study using data from the prospective multicentre AmarCAND2 cohort showed that patients treated with echinocandins were clinically more severely ill and that there was no significant reduction in 28-day mortality in these patients [58]. Similarly, in our study, patients in the echinocandin group were found to be clinically more severely ill. In a study by Reboli et al. [59] on the treatment of invasive candidiasis, anidulafungin was found to be superior to fluconazole, while in another study by Theodora et al. [60], no differences were observed in clinical improvement, microbiological response, and survival between patients receiving micafungin and fluconazole.

A key strength of the present study lies in its capacity to capture the 20-year longitudinal dynamics of candidaemia within an intensive care setting. The limitations of our study are the ambiguity of timing, the occurrence of certain exposures in only a very small number of patients, the inability to retrospectively record physician decisions that guided treatment selection, and the shifts in clinical practice over the 20-year period. Another limitation of this study is that historical isolates collected before 2020 could not be systematically re-evaluated via MALDI-TOF MS. It is worth noting that *Candidozyma auris* was not isolated in our facility throughout the defined study period, as the challenges associated with *Candidozyma auris* emerged institution (not ICU)-wide toward 2024. Furthermore, *Candida haemulonii*, a species historically prone to misidentification as *Candidozyma auris* under biochemical methods, was documented in only one patient during the study span. Regrettably, this isolate could not be subcultured or revitalized for definitive proteomic or genomic validation. In addition to that, the prominence of *Candida parapsilosis* as the second most prevalent pathogen is particularly striking and draws urgent attention to the necessity of restructuring catheter maintenance and insertion practices.

This is a retrospective observational study, and its findings cannot establish causality. All reported associations, including those between clinical factors and severe presentation and between severe presentation and mortality, may be affected by residual confounding. The fluconazole–echinocandin comparison is particularly limited: treatment was selected by clinicians on the basis of species identification and clinical condition, echinocandins were given almost exclusively to patients with non-albicans candidaemia, only 25 patients received an echinocandin, and variables such as calendar period and source control were not available for adjustment. Although propensity score weighting balanced the measured covariates, the E-values (1.31–2.31) show that even modest unmeasured confounding could explain the observed estimates. Therefore, this comparison cannot support the choice of one antifungal agent over the other in clinical practice.

## 8. Conclusions

In conclusion, it was found that patients with higher APACHE-II scores, steroid use or TPN use had a high risk of severe presentation (sepsis or septic shock) associated with candidaemia. While crude mortality rates associated with candidaemia were found to be high, the effect of early antifungal treatment on mortality could not be demonstrated. The higher rate of early antifungal treatment among patients who died may reflect confounding by indication, as treatment was started earlier in more severely ill patients. The presence of severe presentation was strongly associated with 28-day mortality. Because of the observational design and the small number of echinocandin-treated patients, no conclusion can be drawn about the comparative effectiveness of fluconazole and echinocandins, and our results should not be used to guide antifungal selection.

## Figures and Tables

**Figure 1:**
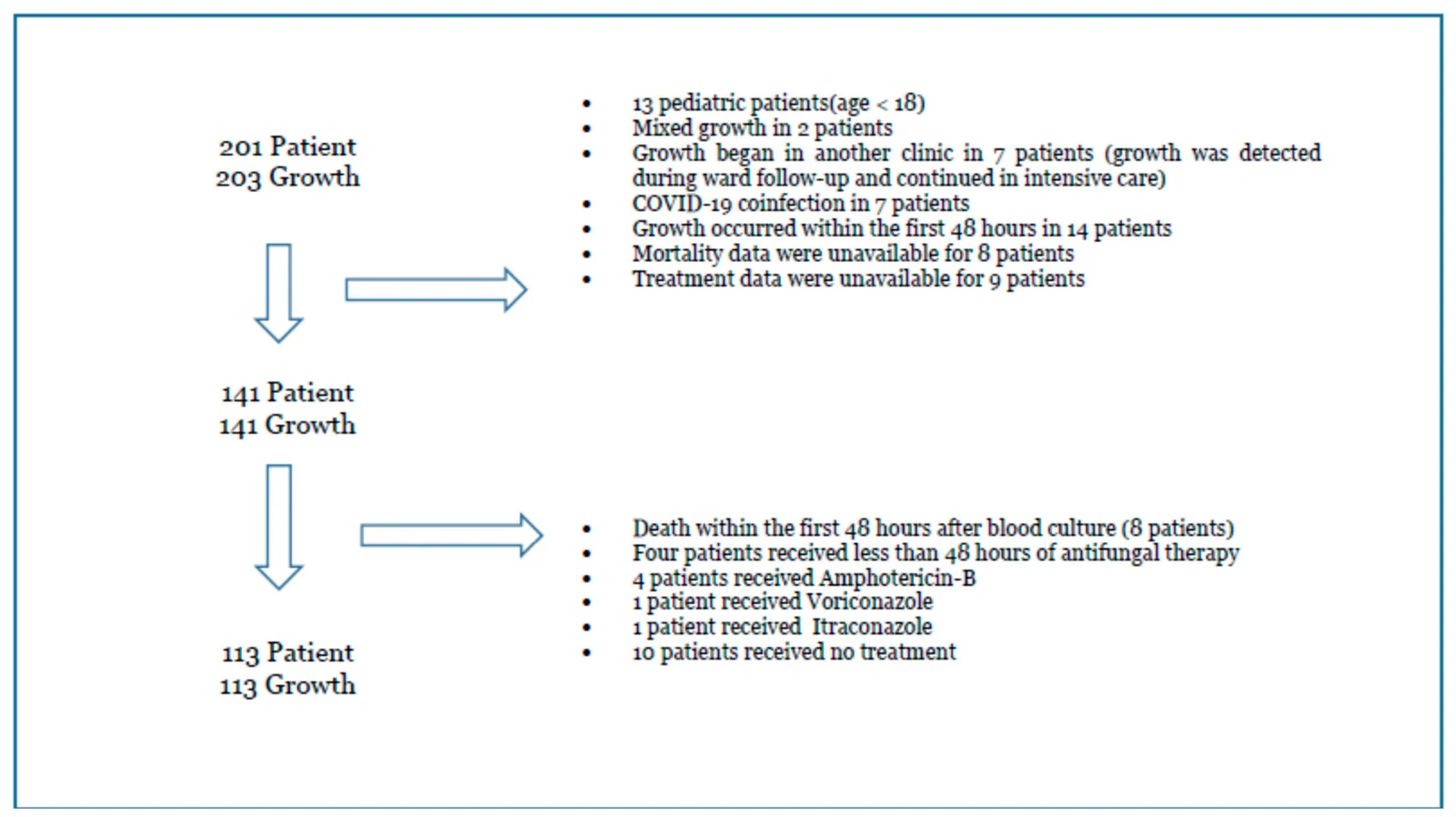
Study design.

**Table 1:** EQUAL Candida Score used in the study.

Diagnosis	Blood culture collection		3
	Species identification		3
	Sensitivity testing		2
	Echocardiographic evaluation		1
	Ophthalmologic evaluation		1
Treatment	Echinocandin therapy		3
	Switch to fluconazole based on sensitivity test results		2
	Treatment for 14 days after first negative follow-up culture		2
	Central venous catheter (CVC) removal * Within the first 24 h after diagnosis (≤24 h) Within 24–72 h after diagnosis		3, 2
Follow-up	Blood cultures should be taken at least once a day until the test is negative.		2
Patients without CVC		Patients with CVC	
Diagnosis	10	Diagnosis	10
Treatment	7	Treatment	10
Follow-up	2	Follow-up	2
Total	19	Total	22

* There is a difference in total points between those with and without CVC because those with CVC receive extra points.

**Table 2:** Demographic, epidemiological, and clinical characteristics of patients with candidaemia.

**Total n (%)**	**141**
Female	70 (49.7%)
Male	71 (50.3%)
Age (years)	56.2 ± 18.4 (19–95)
Total Hospital Stay ^1^ (day)	30.9 ± 37.4 (2–321)
Total ICU stay ^1^ (day)	20.6 ± 34.4 (2–321)
APACHE-II Score On The Day Of ICU Admission	23.7 ± 7.9 (4–46)
EQUAL Candida Score	10.1 ± 4 (6–21)
Concomitant bacteremia n (%) ^2^	26 (18.4)
Breakthrough candidaemia n (%) ^3^	32 (22.7)
TPN n (%)	76 (54)
CRRT n (%)	22 (15)
ECMO n (%)	2 (1.4)
Neutropenia (<500 mm^3^) n (%)	5 (3.5)
Chemotherapy/radiotherapy (CT/RT) (Last 6 months) n (%)	16 (11.3)
**Steroid Use n (%)**	34 (24.1)
Prolonged Steroid Use ^4^	12 (9)
Pulse Steroid Use	0
**Species n (%)**	
*Candida albicans*	55 (39)
*Candida tropicalis*	9 (6.4)
*Candida parapsilosis* species complex	47 (33.4)
*Candida glabrata species komplex* (*Nakaseomyces glabratus*)	23 (16.3)
*Candida kefyr* (*Kluyveromyces marxianus*)	3 (2.1)
*Candida krusei* (*Pichia kudriavzevii*)	1 (0.7)
*Candida haemulonii* (*Candidozyma haemulonii*)	1 (0.7)
*Candida pelliculosa* (*Wickerhamomyces anomalous*)	1 (0.7)
*Candida lusitaniae* (*Clavispora lusitaniae*)	1 (0.7)
**ICU department n (%)**	
Anesthesiology	79 (56)
General Surgery	44 (31.2)
Neurosurgery	3 (2.1)
Thoracic-Cardiovascular Surgery	13 (9.2)
Cardiology	2 (1.4)
**Source of candidaemia n (%)**	
Central venous catheter	32 (22.7)
Abdomen	52 (36.9)
Urinary	2 (1.4)
Primary (unknown)	55 (39)
**The day the blood culture was taken n (%)**	
Sepsis	36 (25.5)
Severe sepsis	44 (31.2)
Septic shock	61 (43.3)
Immunomodulator use ^5^ n (%)	7 (4.9)
At least one comorbidity n (%)	126 (89.3)
At least one immunosuppressive condition ^6^ n (%)	33 (23.4)
Candida colonization in at least one anatomical site n (%)	57 (40.4)
Abdominal surgery (Last 3 months) n (%)	50 (36)
**Comorbidities n (%)**	
Chronic Renal Failure	14 (10)
Diabetes Mellitus	27 (19)
Coronary Artery Disease	40 (28)
Hypertension	32 (22)
Solid Organ Malignancy	28 (20)
Hematologic Malignancy	8 (5.6)
Pancreatitis	3 (2)
Multitrauma	14 (10)
Autoimmune Disease	12 (9)
Asthma-COPD	8 (5.6)
Solid Organ Transplant	4 (3)
Metabolic-Genetic Disease	7 (4)
Liver Failure	5 (3.4)
Cranial Disease	12 (9)
Others ^7^	13 (9.3)
Not receiving treatment	10 (7)
Total Treatment Duration ^8^ (gün)	16.7 ± 11.8 (1–74)
**Antifungal n (%)**	131(100%)
Fluconazole	93 (70.1)
Echinocandin	32 (24.4)
Amphotericin B	4 (3.5)
Others (1 voriconazole, 1 itraconazole)	2(2)
**Overall mortality n (%) ^9^**	
7-day	49 (34.7)
14-day	59 (41.8)
28-day	77 (54.6)
3-month	101 (71.6)
6-month	108 (76.6)

Descriptive statistics are expressed as mean, standard deviation, frequency (n), and percentage (%). CVC: Central venous catheter, ICU: Intensive care unit, TPN: Total parenteral nutrition, CRRT: Continuous renal replacement therapy, ECMO: Extracorporeal membrane oxygenation, CT-RT: Chemotherapy-Radiotherapy, COPD: Chronic obstructive pulmonary disease. ^1.^ The time elapsed until the day the blood culture was taken. ^2.^ Blood culture growths within 48 h before the onset of candidaemia were screened, and concomitant bacterial growths were recorded. ^3.^ Patients who received antifungal therapy for ≥72 h at the onset of candidaemia were in this group. ^4.^ Defined as use of >20 mg/day prednisolone or equivalent steroid for more than three weeks. ^5.^ All immunomodulators other than steroids are included in this group (Tacrolimus, cyclosporine (CsA), methotrexate (MTX), mycophenolate mofetil (MMF), etc.). ^6.^ It was defined as the presence of at least one complicating immunosuppressive condition such as malignancy, autoimmune disease, solid organ transplant, CT-RT, immunomodulator use, and neutropenia. ^7.^ Benign prostatic hyperplasia, urolithiasis, substance abuse. ^8.^ Average treatment duration for 131 patients treated. ^9.^ Overall mortality was assessed for 141 patients.

**Table 3:** Risk factors associated with severe presentation (Severe Sepsis or Septic Shock) in ICU-acquired candidaemia.

	Sepsis	Severe Sepsis or Septic Shock	*p*
**Number (n)**	36	105	
Age (years)	50.4 ± 17.1 (23–95)	58.3 ± 18.6 (19–94)	0.011
**Gender n (%)**			0.885
Female	17 (47)	53 (51)	
Male	19 (53)	52 (49)	
APACHE-II Score On The Day Of ICU Admission	19 ± 7.7 (4–37)	25.3 ± 7.4 (9–46)	<0.001
EQUAL Candida Score	10.8 ± 4.9 (6–21)	10 ± 3.7 (6–17)	0.467
Total Hospital Stay, (day) ^1^	31.7 ± 31 (2–120)	30.7 ± 39 (2–321)	0.733
Total ICU stay, (day) ^1^	17.5 ± 19 (2–74)	21.9 ± 38 (2–321)	0.589
**Concomitant bacteremia n (%) ^2^**	12 (33)	14(13)	0.008
Gram Negative	5 (14)	6 (6)	
Gram Positive	7 (20)	8 (7)	
Breakthrough candidaemia n (%) ^3^	8 (22)	24 (23)	0.937
**ICU department n (%)**			0.239
Anesthesiology	21 (58)	58 (55)	
General Surgery	12 (33)	32 (31)	
Neurosurgery	2 (6)	1 (1)	
Thoracic-Cardiovascular Surgery	1 (3)	12 (11)	
Cardiology	0	2 (2)	
**Source of candidaemia n (%)**	12 (33)	40 (38)	0.616
Abdomen Primary	16 (44)	39 (37)	
Central venous catheter	7 (20)	25 (24)	
Urinary	1 (3)	1 (1)	
**Species n (%)**			0.987
*C. albicans*	14 (39)	41 (39)	
Non-*C. albicans*	22 (61)	64 (61)	
TPN n (%)	12 (33)	64 (61)	0.004
CRRT n (%)	3 (8)	19 (18)	0.164
ECMO n (%)	1 (3)	1 (1)	0.447
Neutropenia n (%)	0	5 (5)	0.329
Chemotherapy/radiotherapy (CT/RT) n (%)	2 (6)	14 (13)	0.360
Abdominal surgery (Last 3 months) n (%)	11 (31)	39 (37)	0.660
Immunomodulator use n (%) ^4^	0	7 (7)	0.191
**Steroid Use n (%)**	4 (11)	30 (29)	0.042
Prolonged Steroid Use ^5^	2 (6)	5 (5)	
Pulse Steroid Use	0	0	
At least one immunosuppressive condition n (%) ^6^	8 (22.2)	25 (23.8)	0.846
Candida colonization in at least one anatomical site n (%)	13 (36)	44 (42)	0.541
**At least one comorbidity n (%)**	31 (86)	95 (91)	0.532
Chronic Renal Failure	1 (3)	13 (12)	0.117
Diabetes Mellitus	5 (14)	22 (21)	0.353
Coronary Artery Disease	3 (8)	37 (35)	0.002
Hypertension	7 (19)	25 (24)	0.589
Solid Organ Malignancy	7 (19)	21 (20)	0.943
Hematologic Malignancy	0	8 (7)	0.114
Pancreatitis	0	3 (3)	0.570
Multitrauma	7 (19)	7 (7)	0.047
Autoimmune Disease	2 (6)	10 (10)	0.731
Asthma-COPD	1 (3)	7 (7)	0.680
Solid Organ Transplant	0	4 (4)	0.572
Metabolic-Genetic Disease	2 (6)	5 (5)	1
Liver Failure	1 (3)	4 (4)	1
Cranial Disease	5 (14)	7 (7)	0.184
Others ^7^	4 (11)	9 (9)	0.739

Descriptive statistics are expressed as mean, standard deviation, frequency (n), and percentage (%). CVC: Central venous catheter, ICU: Intensive care unit, TPN: Total parenteral nutrition, CRRT: Continuous renal replacement therapy, ECMO: Extracorporeal membrane oxygenation, CT-RT: Chemotherapy-Radiotherapy, COPD: Chronic obstructive pulmonary disease. ^1.^ The time elapsed until the day the blood culture was taken. ^2.^ Blood culture growths within 48 h before the onset of candidaemia were screened, and concomitant bacterial growths were recorded. ^3.^ Patients who received antifungal therapy for ≥72 h at the onset of candidaemia were included in this group. ^4.^ All immunomodulators other than steroids are included in this group (Tacrolimus, cyclosporine (CsA), methotrexate (MTX), mycophenolate mofetil (MMF), etc.). ^5.^ Defined as use of >20 mg/day prednisolone or equivalent steroid for more than three weeks. ^6.^ It was defined as the presence of at least one complicating immunosuppressive condition such as malignancy, autoimmune disease, solid organ transplant, CT-RT, immunomodulator use, and neutropenia. ^7.^ Overall mortality was assessed for 141 patients. 28-day mortality was 14% (n = 5) in the sepsis group and 69% (n = 72) in the severe sepsis or septic shock group, *p* < 0.001.

**Table 4:** Risk factors for 28-day mortality in patients with candidaemia.

	Survivors 64 (45.4%)	Non-Survivors 77 (54.6%)	*p*
Age (year)	57.1 ± 18.3 (19–94)	55.5 ± 18.7 (19–95)	0.655
Gender n (%)			0.451
Female	34 (53.1)	36 (46.8)	
Male	30 (46.9)	41 (53.2)	
APACHE-II Score On The Day Of ICU Admission	21.5 ± 7.7 (4–40)	25.4 ± 7.7 (9–46)	0.005
EQUAL Candida Score	12.2 ± 4.2 (6–21)	8.5 ± 3.1 (6–15)	0.040
Total Hospital Stay ^1^ (days)	29.8 ± 30.2 (2–132)	31.8 ± 42.6 (2–321)	0.862
Total ICU stay ^1^ (days)	20.5 ± 23.8 (2–95)	20.7 ± 41.3 (2–321)	0.359
At least one comorbidity n (%)	56 (87.5)	70 (90.9)	0.513
At least one immunosuppressive condition ^2^ n (%)	14(21.9)	19(24.7)	0.695
Candida colonization in at least one anatomical site n (%)	25 (39.1)	32 (41.6)	0.764
Concomitant bacteremia ^3^ n (%)	14 (21.9)	12 (15.6)	0.338
Breakthrough candidaemia ^4^ n (%)	13 (20.3)	19 (24.7)	0.538
TPN n (%)	26 (40.6)	50 (64.9)	0.004
CRRT n (%)	9 (14.1%)	13 (16.9)	0.646
ECMO n (%)	2 (3.1%)	0	0.204
Neutropenia n (%)	0	5 (6.5)	0.063
Chemotherapy/radiotherapy (Last 6 months) n (%)	3 (4.7)	13 (16.9)	0.023
Abdominal surgery (Last 3 months) (%)	22 (34.4)	28 (36.4)	0.806
Steroid Use n (%)	12 (18.8)	22 (28.6)	0.175
Prolonged Steroid Use ^5^	5 (7.8)	7 (9.1)	0.787
Immunomodulator use ^6^ n (%)	3 (4.7)	4 (5.2)	1.000
**The day the blood culture was taken n (%)**			<0.001
Sepsis	31 (48.4)	5 (6.5)	
Severe sepsis or septic shock	33 (51.6)	72 (93.5)	
**ICU department n (%)**			0.752
Anesthesiology	36 (56.3)	43 (55.8)	
General Surgery	21 (32.8)	23 (29.9)	
Neurosurgery	2 (3.1)	1 (1.3)	
Thoracic-Cardiovascular Surgery	5 (7.8)	8 (10.4)	
Cardiology	0	2(2.6)	
**Species n (%)**			0.304
*C. albicans*	22 (34.4)	33 (42.9)	
Non-*C. albicans*	42 (65.6)	44 (57.1)	
**Source of candidaemia n (%)**			0.460
Central venous catheter	16 (25.0)	16 (20.8)	
Abdomen	23 (35.9)	29 (37.7)	
Urinary	2 (3.1)	0	
Primary (unknown)	23 (35.9)	32 (41.6)	
**Comorbidities n (%)**			
Chronic Renal Failure	3 (4.7)	11 (14.3)	0.058
Diabetes Mellitus	14 (21.9)	13 (16.9)	0.453
Coronary Artery Disease	17 (26.6)	23 (29.9)	0.664
Hypertension	16 (25)	16 (20.8)	0.551
Solid Organ Malignancy	11 (17.2)	17 (22.1%)	0.469
Hematologic Malignancy	1 (1.6)	7 (9.1)	0.072
Pancreatitis	2 (3.1)	1 (1.3)	0.591
Multitrauma	11 (17.2)	3 (3.9)	0.009
Autoimmune Disease	4 (6.3)	8 (10.4)	0.380
Asthma-COPD	2 (3.1)	6 (7.8)	0.292
Solid Organ Transplant	2 (3.1)	2 (2.6)	1.000
Metabolic-Genetic Disease	2 (3.1)	5 (6.5)	0.456
Liver Failure	1 (1.6)	4 (5.2)	0.377
Cranial Disease	5 (7.8)	7 (9.1)	0.787
Others ^7^	9 (14.1)	4 (5.2)	0.070
Early antifungal therapy ^8^ (<48 h)	29 (45.3)	49 (63.6)	0.001
**Antifungal ^8^ n (%)**	64 (100.0)	67 (87)	0.329
Fluconazole	49 (76.5)	44 (57.1)	
Echinocandin	14 (21.9)	18 (23.4)	
Amphotericin B	1 (1.6)	3 (3.9)	
Others (one voriconazole, one itraconazole)	0	2 (2.6)	

Descriptive statistics are expressed as mean, standard deviation, frequency (n), and percentage (%). COPD: Chronic obstructive pulmonary disease, CRRT: Continuous renal replacement therapy, ECMO: Extracorporeal membrane oxygenation, ICU: Intensive care unit, TPN: Total parenteral nutrition. ^1.^ The time elapsed until the day the blood culture was taken. ^2.^ It was defined as the presence of at least one complicating immunosuppressive condition such as malignancy, autoimmune disease, solid organ transplant, chemotherapy–radiotherapy, immunomodulator use, and neutropenia. ^3.^ Blood culture growths within 48 h before the onset of candidaemia were screened; concomitant bacterial growths were recorded. ^4.^ Patients who received antifungal therapy for ≥72 h at the onset of candidaemia were included in this group. ^5.^ Defined as use of >20 mg/day prednisolone or equivalent steroid for more than three weeks. ^6.^ All immunomodulators other than steroids are included in this group (tacrolimus, cyclosporine (CsA), methotrexate (MTX), mycophenolate mofetil (MMF), etc.). ^7.^ Benign prostatic hyperplasia, urolithiasis, substance abuse. ^8^. Analyzed among 131 patients who received antifungal therapy.

**Table 5:** Comparison of patients treated with fluconazole or echinocandin.

	Fluconazole	Echinocandin	*p*
**Total**	88 (78%)	25 (22%)	
Age(years)	55.4 ± 17.7 (19–86)	59.9 ± 22.3 (19–95)	0.312
Gender n (%) Female	44 (50)	14 (56)	0.596
Male	44 (50)	11 (44)	
APACHE-II Score On The Day Of ICU Admission	22.3 ± 7.4 (5–42)	23.6 ± 7.6 (4–40)	0.332
EQUAL Candida Score	10.6 ± 4.2 (6–21)	9.8 ± 4 (6–18)	0.505
Total Hospital Stay, (day) ^1^	29.3 ± 30 (2–132)	32.4 ± 29 (2–120)	0.408
Total ICU Stay, (day) ^1^	19.8 ± 23.2 (2–132)	22.4 ± 25 (2–87)	0.720
Breakthrough candidaemia n (%) ^2^	20 (23)	6 (24)	0.894
Concomitant bacteremia n (%) ^3^	19 (22)	5 (20)	0.864
**ICU department n (%)**			0.322
Anesthesiology	49 (56)	10 (40)	
General Surgery	27 (31)	12 (48)	
Neurosurgery	2 (2)	1 (4)	
Thoracic-Cardiovascular Surgery	10 (11)	2 (8)	
Cardiology	0	0	
**Species n (%)**			0.001
*C. albicans*	42 (48)	1 (4)	
Non-*C. albicans*	46 (52)	24 (96)	
**Source of candidaemia n (%)**			0.864
Abdomen	31 (35)	11 (44)	
Primary	33 (38)	8 (32)	
Central venous catheter	22 (25)	6 (24)	
Urinary	2 (2)	0	
**The day the blood culture was taken n (%)**			0.149
Sepsis	31 (35)	5 (20)	
Severe sepsis or septic shock	57 (65)	20 (80)	
TPN n (%)	47 (53)	14 (56)	0.819
CRRT n (%)	5 (6)	10 (40)	0.001
ECMO n(%)	2 (2)	0	1
Neutropenia n(%)	1 (1)	1 (4)	0.395
Chemotherapy/radiotherapy (Last 6 months) n(%)	6 (7)	3 (12)	0.412
Abdominal surgery (Last 3 months) n (%)	32 (36)	11 (44)	0.488
Immunomodulator use n (%) ^4^	4 (5)	3 (12)	0.180
Steroid Use n (%)	16 (18)	9 (36)	0.058
Prolonged Steroid Use n(%) ^5^	6 (7)	2 (8)	1
At least one immunosuppressive condition ^6^	34 (39)	16 (64)	0.024
Candida colonization in at least one anatomical site n (%)	34 (39)	15 (60)	0.057
**At least one comorbidity n (%)**	77(88)	24 (96)	0.296
Chronic Renal Failure	5 (6)	6 (24)	0.014
Diabetes Mellitus	14 (16)	9 (36)	0.028
Coronary Artery Disease	24 (27)	7 (28)	0.943
Hypertension	22 (25)	6 (24)	0.919
Solid Organ Malignancy	16 (18)	6 (24)	0.570
Hematologic Malignancy	3 (3)	2 (8)	0.306
Pancreatitis	3 (3)	0	1
Multitrauma	12 (14)	1 (4)	0.291
Autoimmune Disease	4 (5)	3 (12)	0.180
Asthma-COPD	6 (7)	1 (4)	1
Solid Organ Transplant	3 (3)	1 (4)	1
Metabolic-Genetic Disease	2 (2)	2 (8)	0.212
Liver Failure	1 (1)	1 (4)	0.395
Cranial Disease	7 (8)	3 (12)	0.689
Others ^7^	10 (11)	3 (12)	1
Early antifungal therapy (<48 h)	48 (55)	16 (64)	0.4
14-day mortality n (%)	23 (26)	8 (32)	0.562
28-day mortality n (%)	39 (44)	10 (40)	0.701

Descriptive statistics are expressed as mean, standard deviation, frequency (n), and percentage (%). CVC: Central venous catheter, ICU: Intensive care unit, TPN: Total parenteral nutrition, CRRT: Continuous renal replacement therapy, ECMO: Extracorporeal membrane oxygenation, CT-RT: Chemotherapy-Radiotherapy, COPD: Chronic obstructive pulmonary disease. ^1.^ The time elapsed until the day the blood culture was taken. ^2.^ Patients who received antifungal therapy for ≥72 h at the onset of candidaemia were in this group. ^3.^ Blood culture growth within 48 h before the onset of candidaemia was screened, and concomitant bacterial growth was recorded. ^4.^ All immunomodulators other than steroids are included in this group (Tacrolimus, cyclosporine (CsA), methotrexate (MTX), mycophenolate mofetil (MMF), etc.). ^5.^ Defined as use of >20 mg/day prednisolone or equivalent steroid for more than three weeks. ^6.^ It was defined as the presence of at least one complicating immunosuppressive condition such as malignancy, autoimmune disease, solid organ transplant, CT-RT, steroid use, immunomodulator use, and neutropenia. ^7.^ Benign prostatic hyperplasia, urolithiasis, substance abuse.

**Table 6:** 28-day mortality with echinocandin versus fluconazole. No analysis showed a significant difference, and every confidence interval spans both benefit and harm.

Cohort	Method	Mortality, Fluconazole (%)	Mortality, Echinocandin (%)	OR (95% CI)	Risk Difference, Percentage Points (95% CI)	*p*	E-Value
113	Unweighted	44.3 (39/88)	44.0 (11/25)	0.99 (0.40–2.42)	—	—	—
113	Overlap weights (primary)	49.2	52.1	1.12 (0.37–3.45)	2.9 (−25.1 to 30.9)	0.838	1.31
113	Stabilized IPTW	44.1	63.3	2.18 (0.53–9.03)	19.1 (−14.2 to 52.4)	0.280	2.31
113	Truncated IPTW	44.3	59.5	1.84 (0.49–6.92)	15.2 (−16.8 to 47.1)	0.360	2.06
125	Unweighted	47.3 (44/93)	56.3 (18/32)	1.43 (0.64–3.21)	—	—	—
125	Overlap weights	55.5	63.6	1.40 (0.51–3.81)	8.1 (−15.7 to 31.8)	0.508	1.65
125	Stabilized IPTW	49.6	58.9	1.46 (0.49–4.32)	9.3 (−17.2 to 35.8)	0.493	1.71
125	Truncated IPTW	50.0	58.9	1.44 (0.49–4.21)	9.0 (−17.3 to 35.2)	0.506	1.69

OR > 1 indicates higher mortality with echinocandin. Weighted mortality values are weighted proportions. The 113-patient cohort includes patients who survived and received antifungal therapy for at least 48 h; the 125-patient cohort includes all patients who received fluconazole or an echinocandin as first-line therapy. Truncated IPTW: stabilized weights truncated at the 1st and 99th percentiles. CIs are based on robust standard errors. E-values were calculated for the point estimate using the square-root transformation of the OR for a common outcome; the E-value for the confidence limit was 1.00 in all analyses.

## Data Availability

The corresponding author may share the study’s datasets and analyses with those who request them, provided that their institution permits.

## Supplementary Materials

The following supporting information can be downloaded at: https://www.mdpi.com/article/10.3390/jcm15218597/s1, Table S1: Covariate balance before and after weighting; Table S2: Weight diagnostics.

## Abbreviations

ICU	Intensive care unit
TPN	Total parenteral nutrition
APACHE-II	Acute physiology and chronic health evaluation II
ECMM	European Confederation of Medical Mycology
EQUAL	Quality of clinical candidaemia management
EMB	Eosine Methylen Blue Medium
MALDI TOF MS	Matrix-assisted laser desorption ionisation time of flight mass spectrometry
CLSI	Clinical & Laboratory Standards Institute
