# Supplementary material for: Factors Associated with Severe Presentation and Mortality in ICU-Acquired Candidaemia: A 20-Year Single-Centre Retrospective Cohort
Source: J Clin Med. 2026 Nov 4;15(21):8597. doi: 10.3390/jcm15218597 (PMC13764360; doi:10.3390/jcm15218597)
Supplement: Supplementary file 1 [file jcm-15-08597-s001.zip › jcm-4551910-supplementary.pdf]

Supplementary tables

Supplementary Table S1. Covariate balance before and after weighting

A. Primary cohort (n=113)

Covariate	Fluconazole (n=88)	Echinocandin (n=25)	SMD unweighted	SMD IPTW	SMD truncated IPTW	SMD overlap
Age, years	55.2 ± 18.2	61.4 ± 21.0	0.31	−0.14	−0.12	0.00
Female sex, n (%)	44 (50.0)	14 (56.0)	0.12	−0.43	−0.37	0.00
APACHE-II score	22.2 ± 7.4	24.1 ± 7.3	0.26	0.32	0.27	0.00
Severe sepsis or septic shock, n (%)	58 (65.9)	20 (80.0)	0.32	0.29	0.24	0.00
CRRT, n (%)	5 (5.7)	10 (40.0)	0.88	−0.01	0.03	0.00
Chronic renal failure, n (%)	5 (5.7)	6 (24.0)	0.52	0.89	0.72	0.00
Diabetes mellitus, n (%)	14 (15.9)	9 (36.0)	0.46	−0.11	−0.06	0.00
≥1 immunosuppressive condition, n (%)	34 (38.6)	15 (60.0)	0.43	0.35	0.27	0.00
C. albicans, n (%)	41 (46.6)	1 (4.0)	−1.12	−0.12	−0.30	0.00
Source: abdominal, n (%)	30 (34.1)	11 (44.0)	0.20	−0.20	−0.16	0.00
Source: CVC, n (%)	22 (25.0)	6 (24.0)	−0.02	−0.19	−0.15	0.00

B. Sensitivity cohort (n=125)

Covariate	Fluconazole (n=93)	Echinocandin (n=32)	SMD unweighted	SMD IPTW	SMD truncated IPTW	SMD overlap
Age, years	55.7 ± 18.1	61.8 ± 18.8	0.33	−0.07	−0.06	0.00
Female sex, n (%)	46 (49.5)	16 (50.0)	0.01	−0.21	−0.20	0.00
APACHE-II score	22.6 ± 7.8	25.5 ± 7.5	0.37	0.06	0.08	0.00
Severe sepsis or septic shock, n (%)	63 (67.7)	27 (84.4)	0.39	0.14	0.13	0.00
CRRT, n (%)	6 (6.5)	12 (37.5)	0.80	0.03	0.06	0.00

Covariate	Fluconazole (n=93)	Echinocandin (n=32)	SMD unweighted	SMD IPTW	SMD truncated IPTW	SMD overlap
Chronic renal failure, n (%)	5 (5.4)	7 (21.9)	0.49	0.25	0.24	0.00
Diabetes mellitus, n (%)	14 (15.1)	11 (34.4)	0.45	0.08	0.10	0.00
≥1 immunosuppressive condition, n (%)	36 (38.7)	21 (65.6)	0.55	0.10	0.12	0.00
C. albicans, n (%)	43 (46.2)	4 (12.5)	−0.79	−0.43	−0.45	0.00
Source: abdominal, n (%)	34 (36.6)	14 (43.8)	0.15	−0.08	−0.09	0.00
Source: CVC, n (%)	22 (23.7)	6 (18.8)	−0.12	−0.07	−0.08	0.00

Values are mean ± SD or n (%). SMD: standardized mean difference (echinocandin minus fluconazole; denominator = unweighted pooled SD). Reference category for source: primary/other (urinary source, n=2, merged with primary/other). CRRT: continuous renal replacement therapy; CVC: central venous catheter.

Supplementary Table S2. Weight diagnostics

Cohort	Method	Weight range	ESS, fluconazole	ESS, echinocandin	Largest |SMD|
113	Stabilized IPTW	0.23–7.62	65.6	7.1	0.89
113	Truncated IPTW	0.23–5.48	67.4	9.4	0.72
113	Overlap weights	0.00–0.97	37.8	18.8	0.00
125	Stabilized IPTW	0.26–6.23	60.5	19.3	0.43
125	Truncated IPTW	0.27–5.03	66.4	19.3	0.45
125	Overlap weights	0.01–0.93	44.6	25.2	0.00

ESS: effective sample size, $(\Sigma w)^2 / \Sigma w^2$. In the 113-patient cohort, propensity scores ranged from 0.003 to 0.867 in the fluconazole group and from 0.029 to 0.964 in the echinocandin group.
